# CeNet Omnibus: an R/Shiny application to the construction and analysis of competing endogenous RNA network

**DOI:** 10.1186/s12859-021-04012-y

**Published:** 2021-02-18

**Authors:** Xiao Wen, Lin Gao, Tuo Song, Chaoqun Jiang

**Affiliations:** School of Computer Science and Technology, Xidian University, Xi’an, Shaanxi China

**Keywords:** CeRNA, Shiny application, Network analysis

## Abstract

**Background:**

The competing endogenous RNA (ceRNA) regulation is a newly discovered post-transcriptional regulation mechanism and plays significant roles in physiological and pathological progress. CeRNA networks provide global views to help understand the regulation of ceRNAs. CeRNA networks have been widely used to detect survival biomarkers, select candidate regulators of disease genes, and predict long noncoding RNA functions. However, there is no software platform to provide overall functions from the construction to analysis of ceRNA networks.

**Results:**

To fill this gap, we introduce CeNet Omnibus, an R/Shiny application, which provides a unified framework for the construction and analysis of ceRNA network. CeNet Omnibus enables users to select multiple measurements, such as Pearson correlation coefficient (PCC), mutual information (MI), and liquid association (LA), to identify ceRNA pairs and construct ceRNA networks. Furthermore, CeNet Omnibus provides a one-stop solution to analyze the topological properties of ceRNA networks, detect modules, and perform gene enrichment analysis and survival analysis. CeNet Omnibus intends to cover comprehensiveness, high efficiency, high expandability, and user customizability, and it also offers a web-based user-friendly interface to users to obtain the output intuitionally.

**Conclusion:**

CeNet Omnibus is a comprehensive platform for the construction and analysis of ceRNA networks. It is highly customizable and outputs the results in intuitive and interactive. We expect that CeNet Omnibus will assist researchers to understand the property of ceRNA networks and associated biological phenomena. CeNet Omnibus is an R/Shiny application based on the Shiny framework developed by RStudio. The R package and detailed tutorial are available on our GitHub page with the URL https://github.com/GaoLabXDU/CeNetOmnibus.

## Background

MicroRNAs are ~ 22nt small non-coding RNAs, which can bind to microRNA response elements (MREs) on target RNA sequences (e.g. mRNAs, lincRNAs, pseudogenes, and circle RNAs) through the RNA-induced silencing complex (RISC) and lead to transcripts degraded or translation repressed [1–3]. Different transcripts with the same MRE can regulate each other via competitively binding shared microRNAs, which are called competing endogenous RNAs (ceRNAs). The ceRNA regulatory is a new kind of post-transcriptional regulation mechanism and is also considered the “Rosetta Stone of a hidden RNA language” [4, 5]. It has been demonstrated that the complex crosstalk among ceRNAs involves numerous physiological and pathological progress [6–9], such as cell proliferation, differentiation, invasion, and metastasis [10–12]. Because individual microRNAs can target multiple transcripts and the same transcript may contain MREs of different microRNAs, there are giant and complicated networks in cells between microRNAs and their target transcripts, as well as between ceRNAs themselves. An increasing number of studies about ceRNAs and ceRNA networks have been published in the past few years [13, 14], which are widely used to detect survival biomarkers, select candidate regulators of disease genes, and predict long noncoding RNA functions [15].

As the result of the huge scale of the ceRNA networks, computational methods have become efficient approaches to the construction of the ceRNA networks [13, 16]. Current methods are developed based on two basic principles: (1) ceRNA pairs should share a sufficient number of microRNAs; and (2) ceRNA pairs should be co-expressed. For the first measurement, the hyper-geometric test is used to evaluate the enrichment significance of microRNAs of a ceRNA pair. For the second one, statistical indexes can be categorized into two classes: (1) the pair-wised correlation, such as Pearson correlation coefficient (PCC) and mutual information (MI); and (2) partial associated correlation, such as sensitivity correlation (SI) [17], multiple sensitivity correlation [18] and conditional mutual information (CMI) [19]. A part from those methods, Zhang et al. [20] proposed LncmiRSRN to construct a lncRNA-mRNA ceRNA network via estimating the causal effects of lncRNAs on mRNAs with the IDA method [21], where IDA refers to the intervention calculus when the DAG is absent. Recently, we proposed that liquid association (LA) is another new measurement for the identification of ceRNA pairs [22], where LA can be used to estimate the correlation sensitivity of ceRNAs to microRNAs. Besides those computational methods, researchers have established a set of ceRNA databases, such as lnCeDB [23], LncCeRBase [24], miRSponge [25], LncACTdb [26] and PceRBase [27].

Although there has been such great progress in ceRNA, there is no software platform to provide overall functions to construct and analyze the ceRNA networks. In this study, we developed CeNet Omnibus, an R/Shiny based application, which achieves a unified framework to construct and analyze ceRNA network. CeNet Omnibus intends to cover comprehensiveness, high efficiency, high expandability, and user customizability. CeNet Omnibus integrates complete functions for the construction and analysis of ceRNA networks. In order to accommodate to the different usage scenarios, CeNet Omnibus allows users to define some functions and set parameters in different situations. With the support of the R/Shiny framework, CeNet Omnibus offers a web-based user-friendly interface for users to obtain the output intuitionally. We expect that CeNet Omnibus will become an efficient and convenient tool to investigate the ceRNA networks for researchers, especially those, who are not familiar with programming.

## Implementation

CeNet Omnibus is implemented with R programming language (Release 3.6) for the most parts of computation. For some specific functions, we use Java (Version 1.7) codes to improve efficiency. The web-based user interface is developed based on the Shiny framework (Version 1.4) from RStudio, as well as a set of associated packages, such as shinydashboard (Version 0.7), shinyWidgets (Version 0.5). Network construction and analysis are based on igraph package (Version 1.2.4), while the visualization of networks depends on javascript plugin Cytoscape.js (Version 3.13) and visNetwork package (Version 2.0). Other dependency packages are listed on the homepage of CeNet Omnibus.

**Fig. 1:**
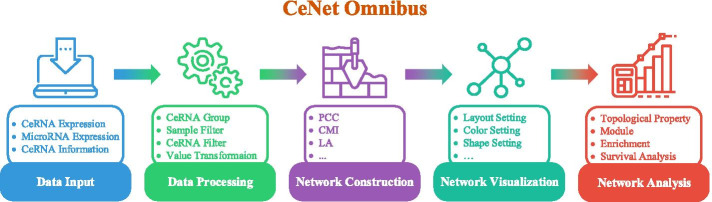
The framework of CeNet Omnibus

CeNet Omnibus consists of five components, including data input, data processing, network construction, network visualization, and network analysis. Figure 1 illustrates the framework of CeNet Omnibus.

### Data input

Users are expected to upload the expression profiles of candidate ceRNAs, the expression profiles of microRNAs, microRNA- candidate ceRNA interaction data, and candidate ceRNA information as inputs before the further operation. All these data should be plain text delimited by tab, comma, space, semicolon, or any other practicable marks. The expression profiles of candidate ceRNA and microRNA should have sample names. The candidate ceRNA and microRNA symbols should meet the same standard in all these files. In addition to direct uploading, we also provide an interface for users to obtain candidate ceRNA information from Ensembl database [28] with the support of biomaRt [29] package, which enables collect large amounts of data in a uniform way.

### Data processing

At the beginning of data processing, the program will automatically obtain three symbol sets, including (1) candidate ceRNAs symbols that are available in all of candidate ceRNA expression profiles data, microRNA- candidate ceRNA interaction data and candidate ceRNA information data, (2) microRNA symbols available in both microRNA expression profiles data and microRNA- candidate ceRNA interaction data, and (3) sample names available in expression profiles of both candidate ceRNAs and microRNAs.

Considering that there are significant differences between the expression levels of different RNA types and that users may want to investigate a part of candidate ceRNA relationships in the datasets, users are allowed to divide candidate ceRNAs into different groups to filter non-expressed RNAs or construct a ceRNA subnetwork. For example, the candidate ceRNAs can be divided into the “Noncoding” RNAs group and the “Coding” RNAs group according to their biotypes.

Users are allowed to remove low-quality samples. For this aim, users are required to provide the thresholds for the detected microRNAs and candidate ceRNAs respectively. Then, CeNet Omnibus plots the histograms of the detected RNAs ratios of samples. Users may determine how many samples should be remained based on the histograms. Furthermore, to remove the non-expressed RNAs, users should input the minimal expression levels of microRNAs and each candidate ceRNA group respectively. Then, CeNet Omnibus creates the histograms of the expressing sample ratios of RNAs. Users may also determine how many RNAs should be remained based on the histograms.

Finally, users can transform the expression values of microRNAs and candidate ceRNAs. We provide the log transformation and the normalization transformation. The two transformations can be executed at the same time, in the order of log transformation first and normalization the next. We have defined two specific normalization functions, which are min–max scaling and z-score scaling. Besides, users can define the normalization function themselves.

### Network construction

The construction of ceRNA network is associated with the identification of ceRNA pairs. CeNet Omnibus provides five different measurements for the detection of ceRNA pairs, including shared microRNA enrichment significance (MS), Pearson correlation coefficient (PCC), mutual information (MI), conditional mutual information (CMI), and liquid association (LA). MS is used to test if a candidate ceRNA pair shares a sufficient number of microRNAs. LA is used to estimate the correlation sensitivity of candidate ceRNAs to shared microRNAs. PCC, MI, and CMI are all used to evaluate the levels of co-expression, where PCC can evaluate the linear correlation, while MI and CMI can evaluate the non-linear correlation.

For a given candidate ceRNA pair (e.g., $R_{1}$ and $R_{2}$), the MS is defined as:

$$MS(R_{1} ,R_{2} ) = 1 - \sum\limits_{i = 0}^{t - 1} {\frac{{\left( {\begin{array}{*{20}c} {T_{1} } \\ i \\ \end{array} } \right) \times \left( {\begin{array}{*{20}c} {Q - T_{1} } \\ {T_{2} - i} \\ \end{array} } \right)}}{{\left( {\begin{array}{*{20}c} Q \\ {T_{2} } \\ \end{array} } \right)}}}$$

where $Q$ is the total number of considered microRNAs, $T_{1}$ is the number of microRNAs targeting $R_{1}$, $T_{2}$ is the number of microRNAs targeting $R_{2}$, and $t$ is the number of microRNAs targeting both $R_{1}$ and $R_{2}$.

Let $e_{{R_{1} }}$ and $e_{{R_{2} }}$ represent the expression of $R_{1}$ and $R_{2}$ respectively, and the PCC of $R_{1}$ and $R_{2}$ is defined as:

$$PCC(R_{1} ,R_{2} ) = \frac{{E[(e_{{R_{1} }} - E(e_{{R_{1} }} )) \times (e_{{R_{2} }} - E(e_{{R_{2} }} ))]}}{{\sqrt {Var(e_{{R_{1} }} ) \times Var(e_{{R_{2} }} )} }},$$

where $E(\cdot)$ and $Var(\cdot)$ represent the expectation and variance of a random variable respectively.

Let $disc(e_{{R_{1} }} )$ and $disc(e_{{R_{2} }} )$ represent the discretized expression of $R_{1}$ and $R_{2}$, the MI of $R_{1}$ and $R_{2}$ is defined as:

$$MI(R_{1} ,R_{2} ) = \sum\limits_{{r_{1} \in disc(e_{{R_{1} }} )}} {\sum\limits_{{r_{2} \in disc(e_{{R_{2} }} )}} {p_{{R_{1} ,R_{2} }} (r_{1} ,r_{2} )\log \frac{{p_{{R_{1} ,R_{2} }} (r_{1} ,r_{2} )}}{{p_{{R_{1} }} (r_{1} )p_{{R_{2} }} (r_{2} )}}} } ,$$

where $p_{{R_{1} ,R_{2} }} (r_{1} ,r_{2} )$ is the joint probability distribution of $R_{1}$ and $R_{2}$, $p_{{R_{1} }} (r_{1} )$ and $p_{{R_{2} }} (r_{2} )$ are the marginal distributions of $R_{1}$ and $R_{2}$ respectively.

Similarly, let $R{}_{mi}$ indicate the shared microRNAs between $R_{1}$ and $R_{2}$, $e_{{R_{mi} }}$ represent the sum of the expression profile of $R{}_{mi}$, and $disc(e_{{R_{mi} }} )$ represent the discretized value of $e_{{R_{mi} }}$. The CMI of $R_{1}$ and $R_{2}$ is defined as:

$$\begin{aligned} CMI(R_{1} ;R_{2} |R_{{mi}} ) & = \sum\limits_{{r_{{mi}} \in disc(e_{{R_{{mi}} }} )}} {\sum\limits_{{r_{1} \in disc(e_{{R_{1} }} )}} {\sum\limits_{{r_{2} \in disc(e_{{R_{2} }} )}} {p_{{R_{1} ,R_{2} ,R_{{mi}} }} (r_{1} ,r_{2} ,r_{{mi}} )} } } \\ & \quad \quad \log \frac{{p_{{R_{{mi}} }} (r_{{mi}} )p_{{R_{1} ,R_{2} ,R_{{mi}} }} (r_{1} ,r_{2} ,r_{{mi}} )}}{{p_{{R_{1} ,R_{{mi}} }} (r_{1} ,r_{{mi}} )p_{{R_{2} ,R_{{mi}} }} (r_{2} ,r_{{mi}} )}} \\ \end{aligned}$$

where $p_{{R_{1} ,R_{2} ,R_{mi} }} (r_{1} ,r_{2} ,r_{mi} )$ is the joint probability distribution of $R_{1}$, $R_{2}$, and $R{}_{mi}$.

The LA of $R_{1}$ and $R_{2}$ is defined as:

$$LA(R_{1} ,R_{2} |R_{mi} ) = \frac{{\sum\limits_{i = 1}^{N} {z_{i} (e_{{R_{1} }} ) \times z_{i} (e_{{R_{2} }} ) \times norm_{i} (e_{{R_{mi} }} )} }}{N},$$

where $N$ is the total number of samples, $z_{i} (e_{{R_{1} }} )$ and $z_{i} (e_{{R_{2} }} )$ are the expression of $R_{1}$ and $R_{2}$ normalized by z-score transformation in *i*th sample respectively, and $norm_{i} (e_{{R_{mi} }} )$ is the *i*th value of the expression of $R{}_{mi}$ normalized via Van der Waerden’s method [30].

CeNet Omnibus also allows users to define new measurements for the detection of ceRNA pairs. Users can select one or a set of measurements to detect ceRNA pairs according to their situation.

To improve the efficiency of the calculation, CeNet Omnibus can calculate these measurements parallelly with the parallel package (Version 3.6). In addition, users can only calculate a part of relations in the datasets depending on the results of gene grouping in Data Processing Section.

After the calculation of selected measurements, CeNet Omnibus will create the density plots for each measurement. Users can select a specific threshold for each computed measurement according to their situation. CeNet Omnibus will collect interactions, which are satisfied with all the thresholds, to construct networks. After the construction of the ceRNA network, CeNet Omnibus will summarize the basic information of the network, including the number of connected nodes, isolated nodes, edges, and connected components.

### Network visualization

Users can use the network visualization component to view the overview structure of the constructed ceRNA network. Users are able to modify the layout of the network, the size, color, and shape of specific nodes, as well as the displayed symbols of nodes. Users can also search for nodes they are interested in for the next analysis.

### Network analysis

For further analyzing the ceRNA network, CeNet Omnibus supplies a one-stop solution, including network topological property analysis, network module detection, gene enrichment, and survival analysis.

In the part of network topological property analysis, CeNet Omnibus provides four topological properties of nodes, which include degree, betweenness, clustering coefficient, and closeness, as well as an topological property of edges–edge betweenness. The analysis of network topological properties can help users to find the important nodes and edges in the ceRNA networks for further analysis.

In the part of network module detection, CeNet Omnibus embeds the popular module detection algorithms, such as Louvain method [31], MCL [32], MCODE [33], and EPCA [34]. Because some algorithms require parameters, CeNet Omnibus allows users to test the performance of different parameters, and it will give a report, including the count of modules, the modularity of modules, the average density of the modules, the modules size distribution, the edge counts of modules distribution, and the modules density distribution, to help users determine the final parameters. The microRNAs associated with edges in modules are considered as the module associated microRNAs, and these microRNAs are also listed in the results of network modules. CeRNAs may form dense subnetworks to regulate each other, and current studies have suggested that the ceRNA modules may be potential biomarkers for cancer therapy. These results may be used for further analysis.

For further understanding the biological and medical principles in the ceRNA networks, CeNet Omnibus provides users the functions of enrichment analysis and survival analysis. The current version has integrated the functional enrichment with g:Profiler [35, 36]. Besides, users can input user-defined datasets for other enrichment analysis. Furthermore, the current version has integrated two survival analysis models to investigate the relationships between ceRNAs and disease therapy. Kaplan–Meier survival estimator is used to study the effect of a single factor on survival probability, while the Cox proportional hazards regression model can estimate multiple factors simultaneously.

## Results

### Comparison with related package

CeRNA networks are effective tools to study the ceRNA regulation. However, there are not enough available computational tools to help researchers construct and analyze the ceRNA networks. We surveyed the published computational tools, and only found the CeRNASeek [37], which is an R package to identify and analyze ceRNA-ceRNA interactions. Comparing with CeRNASeek, CeNet Omnibus is more comprehensive, more expandable, and more user-friendly.

CeNet Omnibus and CeRNASeek are both R-based packages. Different from CeRNASeek, CeNet Omnibus provides a user-friendly web-based interface to eliminate the gap between users and programming. CeNet Omnibus provides comprehensive toolkits, from data processing to network analysis. However, CeRNASeek depends on users to input processed data and lacks the analysis of the network properties. Finally, CeNet Omnibus uses the parallel computing technique to speed up the calculation, and it provides interfaces to expand application scenarios. The comparison details are shown in Table 1.

**Table 1:** The comparison between CeNet Omnibus and CeRNASeek

Items		CeNet Omnibus	CeRNASeek
Environment	Language	R	R
	Operation interface	web-base	command line
Functionality	Data processing	Yes	No
	Network Construction	PCC, MS, MI, CMI, LA, and self define	Ratio, HyperT, HyperC, SC, CMI, and Cernia
	Network Visualization	Yes	Yes
	Network topological Property	Yes	No
	Network module	Yes	No
	Enrichment analysis	Yes	Yes
	Survival analysis	Yes	Yes
Feature	Usability	GUI with document tutorial	R function with document tutorial
	Efficiency	Yes	No
	Expandability	Yes	No

### Case study: K562 single-cell microRNA-mRNA co-sequencing data

To examine the performance of CeNet Omnibus, we applied CeNet Omnibus on single-cell microRNA-mRNA co-sequencing data from [38]. The data set contains 23,284 mRNAs/noncoding RNAs FPKM values and 2822 mature microRNAs log2-transformed miRNA expression of 19 K562 single cells. The interactions between ceRNAs and microRNAs are downloaded from mirwalk2.0 database [39]. To obtain the gene biotypes, we downloaded the gene information from Ensembl database [28]. The sample data is shown in Additional file 1: Table S1.

**Fig. 2:**
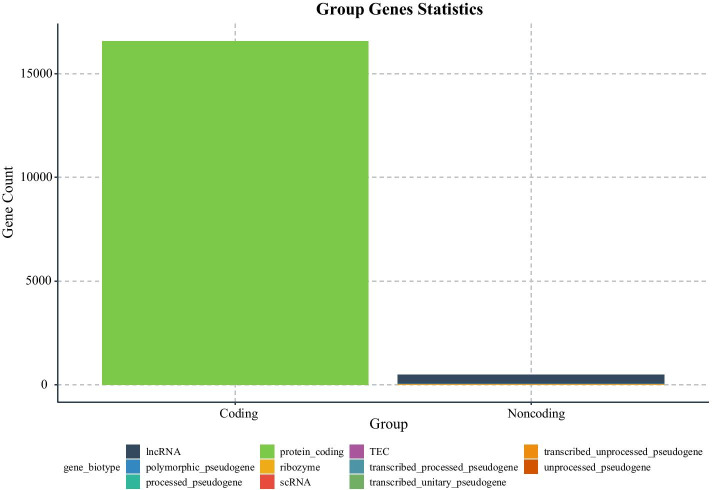
The statistics of genes in different groups

After uploading the four data files into CeNet Omnibus, the program obtains 17,058 valid mRNAs/noncoding RNAs, and 2532 valid microRNAs of 19 valid samples. We group the mRNAs/noncoding RNAs according to gene biotype attribution in gene information files. We put RNAs, whose biotypes are “protein_coding”, into the “Coding” group, while other RNAs are put into the “Noncoding” group. The gene count statistics are shown in Fig. 2.

**Fig. 3:**
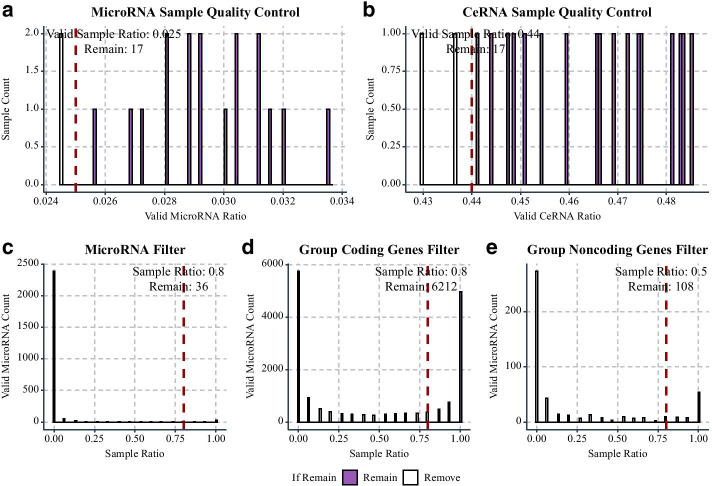
Remove low-quality samples in the expression profiles and non-expressed RNAs. **a** The histogram of the detected microRNA ratio of each sample. **b** The histogram of the detected ceRNAs ratio of each sample. **c** The histogram of the expressing sample ratio of each microRNA. **d** The histogram of the expressing sample ratio of each “Coding” RNAs. **e** The histogram of the expressing sample ratio of each “Noncoding” RNAs

We use log2(1e-4) and 1 as the thresholds of detected microRNAs and mRNAs/noncoding RNAs samples respectively, and remove two samples with the lowest detected RNA ratios for each data set (Fig. 3a, b). Then, we obtain 15 high-quality samples for the next step. To exclude lowly expressed microRNAs, we only retain microRNAs with expression value > log2(2e-4) in > 80% of cells (Fig. 3c). For RNAs in group “Coding”, we retain RNAs with expression value > 1 in > 80% of cells, while for RNAs in group “Noncoding”, we retain RNAs with expression value > 0.5 in > 50% of cells (Fig. 3d, e). Finally, we obtain 36 microRNAs, 6212 “Coding” RNAs, and 108 “Noncoding” RNAs to construct ceRNA networks. The FPKM values of “Coding” and “Noncoding” RNAs are log2-transformed.

**Fig. 4:**
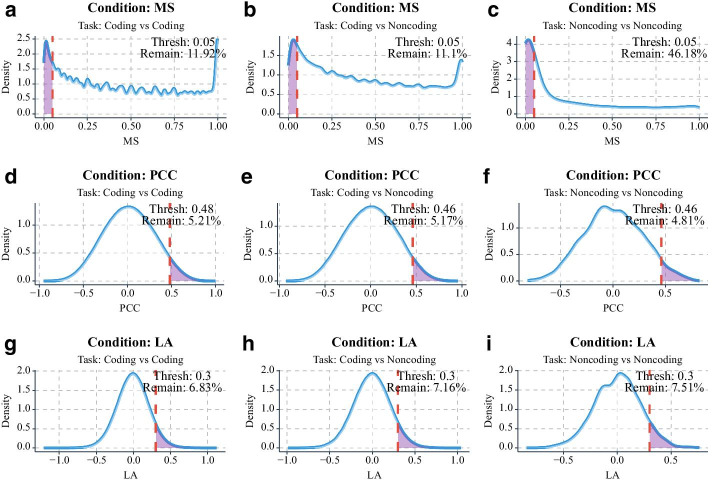
Density plots of the computed measurements of different group pairs. **a**–**c** Density plots of microRNA enrichment significance. **d**–**f** Density plots of Pearson correlation coefficient, **g**–**i** Density plots of liquid association

As CeNet Omnibus provides five different measurements for constructing ceRNA networks, here we use MS, PCC, and LA for the network construction. We calculate these three measurements for each RNA group pair, and the density plots are shown in Fig. 4. We choose the thresholds for each measurement as shown in Table 2. Finally, we obtain a ceRNA network with 3695 nodes, 16,879 edges, and 42 connected components.

**Table 2:** The thresholds used of MS, PCC and LA for each group pair

Measurement	Group pair	Direction	Thresh
MS	Coding versus Coding	<	0.05
	Coding versus Noncoding		
	Noncoding versus Noncoding		
PCC	Coding versus Coding	>	0.48
	Coding versus Noncoding	>	0.46
	Noncoding versus Noncoding	>	0.46
LA	Coding versus Coding	>	0.3
	Coding versus Noncoding		
	Noncoding versus Noncoding		

**Fig. 5:**
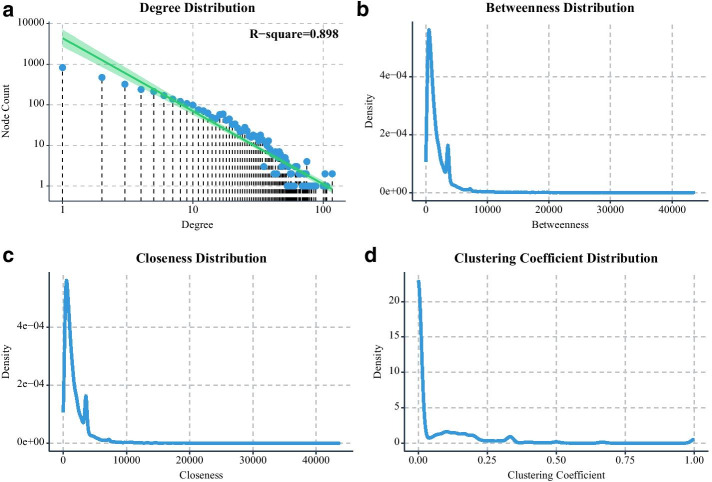
The distribution of node degree (**a**), edge betweenness (**b**), node closeness (**c**), and node clustering coefficient (**d**)

**Fig. 6:**
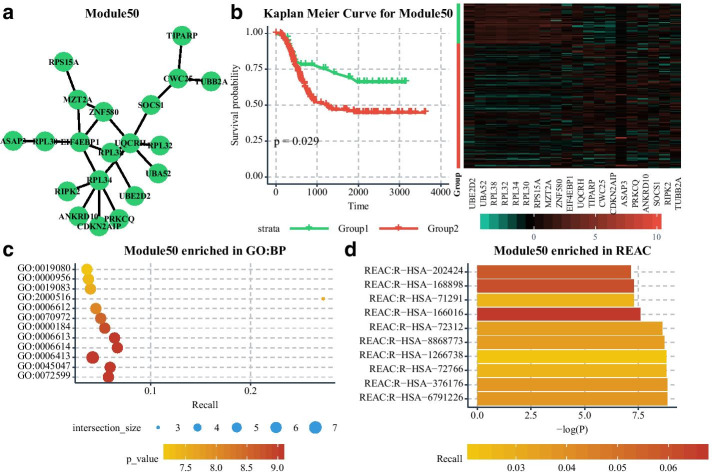
The analysis of Module50. **a** The topological structure of Module50. **b** The Kaplan–Meier curve for Module50 and the patient clustering with Module50. **c**, **d** The GO: BP and REACTOME enrichment terms of Module50

We use the network analysis component to analyze the constructed network. Figure 5 shows a part of topological properties distribution of the network, and the specific topological properties of the partial nodes are shown in Additional file 2: Table S2. We use the Louvain method [31] to detect network modules, and the whole module information is listed in the Additional file 3: Table S3. We use Module50 (Fig. 6a) as an example for further analysis. The GO: BP and REACTOME enrichment terms of Module50 are shown in Fig. 6c, d, and Additional file 4: Table S4. We find Module50 is highly associated with RNA transcript and translation. Furthermore, we download the expression profiles and clinical information of 155 acute myeloid leukemia (AML) patients from TCGA to evaluate if genes of Module50 can be a module biomarker to predict patient prognostic. As shown in Fig. 6b, the patient group with lower expressions of ZNF580, RPL34, RPL30, RPS15A, RPL32, RPL38, and UBA52 has relatively worse prognostic than the group with higher expression.

## Conclusion

CeNet Omnibus is a comprehensive platform for the construction and analysis of ceRNA networks. It provides a friendly-used framework to process uploaded data, apply different strategies to construct ceRNA networks, and investigate the biological and medical principles in the networks. It is highly customizable and outputs the results in intuitive and interactive. In testing on our data, our platform successfully constructed a single-cell ceRNA network and detected a prognostic module biomarker with abundant biological functions. Given its design as an open-source Shiny application with a web-based interface, CeNet Omnibus is an important tool for researchers to systematically study the properties of different ceRNA networks and their effects in different biological progresses.

## Availability and requirements

- Project name: CeNet Omnibus
- Project home page: https://github.com/GaoLabXDU/CeNetOmnibus
- Operating system(s): Platform independent
- Programming language: R
- Other requirements: Java 1.7 or higher, web browsers, internet connectivity
- License: MIT
- Any restrictions to use by non-academics: None

## Supplementary Information

**Additional file 1: Table S1.** The data sample uploaded to the platform.

**Additional file 2: Table S2.** The information of the topological properties of a part of nodes.

**Additional file 3: Table S3.** The basic information of modules.

**Additional file 4: Table S4.** The enrichment terms of Module50.

## Data Availability

All datasets used in this study have been previously published. The single cell RNA and microRNA expression profiles are available in Gene Expression Omnibus repository [https://www.ncbi.nlm.nih.gov] under GEO series GSE114071. The data of AML patients is available in GDC [https://portal.gdc.cancer.gov/].

## Abbreviations

AML: Acute myeloid leukemia
ceRNA: Competing endogenous RNA
CMI: Conditional mutual information
FPKM: Fragments per kilobase of transcript per million
LA: Liquid association
MI: Mutual information
MRE: MicroRNA response element
MS: Shared microRNA enrichment significance
PCC: Pearson correlation coefficient
RISC: RNA-induced silencing complex
TCGA: The Cancer Genome Atlas
